# Supplementary material for: Mapping the evolution of fertility support policies in China: A content and instrumental analysis
Source: PLoS One. 2025 Oct 9;20(10):e0332137. doi: 10.1371/journal.pone.0332137 (PMC12510515; doi:10.1371/journal.pone.0332137)
Supplement: S1 Appendix — (ZIP) [file pone.0332137.s001.zip › S1 Appendix. 226 original policy documents/68-国务院关于儿童健康促进工作情况的报告(FBM-CLI-2-5127968).doc]

国务院关于儿童健康促进工作情况的报告

**发布部门：** [国务院](https://www.pkulaw.com/law/chl?Aggs.IssueDepartment=5)

**发布日期：**2022.06.21

**实施日期：**2022.06.21

**时效性：** [现行有效](https://www.pkulaw.com/law/chl?Aggs.TimelinessDic=01)

**效力级别：** [国务院规范性文件](https://www.pkulaw.com/law/chl?Aggs.EffectivenessDic=XC0203)

**法规类别：** [老少妇幼残保护](https://www.pkulaw.com/law/chl?Aggs.Category=01604)

国务院关于儿童健康促进工作情况的报告
--2022年6月21日在第十三届全国人民代表大会常务委员会第三十五次会议上
国家卫生健康委员会主任 马晓伟

全国人民代表大会常务委员会：
　　按照全国人大常委会安排，受国务院委托，就儿童健康促进工作情况报告如下，请审议。
　　党中央、国务院高度重视儿童健康促进工作。习近平总书记指出，儿童健康事关家庭幸福和民族未来，在全国卫生与健康工作大会上强调要重视少年儿童健康，主持中央全面深化改革领导小组会议审议《关于加强儿童医疗卫生服务改革与发展的意见》，对做好儿童青少年近视和肥胖防控、心理健康服务、缓解儿童医疗服务资源短缺问题、幼儿园和中小学卫生与健康工作等多次作出重要指示批示。李克强总理多次作出重要批示，强调要推进儿童健康服务的优先供给，完善儿童医疗卫生服务网络，深入实施健康儿童计划。孙春兰副总理多次主持召开会议，研究重要政策措施，部署推进重点工作。各地各有关部门深入贯彻习近平总书记重要指示精神，认真落实党中央、国务院决策部署，不断完善政策，狠抓工作落实。
　　**一、**主要工作和成效
　　（一）完善儿童健康促进法规政策。全国人大以[宪法](https://www.pkulaw.com/chl/7c7e81f43957c58bbdfb.html)为依据，不断完善儿童健康法律体系，制定公布[基本医疗卫生与健康促进法](https://www.pkulaw.com/chl/22a006a09f3565e4bdfb.html)，明确规定国家发展妇幼保健事业，建立健全妇幼健康服务体系。两次修订[人口与计划生育法](https://www.pkulaw.com/chl/d560ce000fba464cbdfb.html)，不断强化提高出生人口素质，加强母婴保健和婴幼儿照护服务。修订[未成年人保护法](https://www.pkulaw.com/chl/883cecd7fe38636dbdfb.html)，坚持最有利于未成年人的原则，加强未成年人全方位保护。修订[药品管理法](https://www.pkulaw.com/chl/5374e2b0e2451f75bdfb.html)，进一步明确鼓励儿童用药品的研制和创新，对儿童用药予以优先审评审批。在[民法典](https://www.pkulaw.com/chl/aa00daaeb5a4fe4ebdfb.html)、[传染病防治法](https://www.pkulaw.com/chl/b34b627b643cd519bdfb.html)、[疫苗管理法](https://www.pkulaw.com/chl/2a0405a853c122f3bdfb.html)、[食品安全法](https://www.pkulaw.com/chl/ac73cbe3ed780d7fbdfb.html)、[家庭教育促进法](https://www.pkulaw.com/chl/99c5277887339564bdfb.html)、学前[教育法](https://www.pkulaw.com/chl/20f59ac63eeb54a8bdfb.html)、[反家庭暴力法](https://www.pkulaw.com/chl/ac57ba1df4413457bdfb.html)等法律制修订中，均强化儿童健康促进有关规定。
　　国务院及各有关部门认真贯彻落实党中央决策部署和相关法律要求，坚持依法行政，全力推进儿童健康促进工作。制定实施四个周期中国儿童发展纲要，针对儿童健康关键问题，明确主要目标和策略措施。在《“健康中国2030”规划纲要》中，明确到2030年婴儿死亡率和5岁以下儿童死亡率控制在5.0‰和6.0‰，提出实施健康儿童计划。将儿童健康纳入《[国务院关于实施健康中国行动的意见](https://www.pkulaw.com/chl/542548642f0c86bfbdfb.html)》等重要文件，建立健全工作推进机制。
　　（二）健全儿童健康促进保障机制。一是推动建立党委领导、政府主导、多部门分工合作、全社会共同参与的工作机制。强化国务院妇女儿童工作委员会作用，成立国务院未成年人保护工作领导小组，统筹推进儿童健康事业发展。二是建立持续的儿童健康投入保障机制。将预防接种、儿童健康管理、儿童中医药健康管理纳入国家基本公共卫生服务项目免费向0-6岁儿童提供。中央和地方各级财政持续加大投入，2012年以来，国家基本公共卫生服务项目经费人均财政补助标准从25元提高到2020年的74元，中央和地方各级财政共投入5934.85亿元，根据安排，2021年人均财政补助标准再提高5元。在打赢脱贫攻坚战中统筹加大对贫困地区儿童健康投入，印发《国家贫困地区儿童发展规划（2014-2020年）》，强化贫困地区儿童健康促进。三是建立覆盖包括儿童在内的全民基本医疗保障制度。强化基本医疗保险、大病保险与医疗救助三重保障功能，促进各类医疗保障政策互补衔接，稳步提高包括儿童在内的广大城乡居民整体医疗保障水平。加快儿童药品纳入医保，儿童专用药和适用药占国家医保目录内药品种类的20.13%，经谈判新进入目录的34个独家儿童用药品平均降价55.6%。四是建立残疾儿童康复救助制度。连续制定实施两周期国家残疾预防行动计划，开展出生缺陷和发育障碍致残防控行动。印发《[关于建立残疾儿童康复救助制度的意见](https://www.pkulaw.com/chl/c3753aee45486b5dbdfb.html)》，为符合条件的0-6岁视力、听力、言语、肢体、智力等残疾儿童和孤独症儿童进行康复救助。2012年以来，累计救助0-6岁残疾儿童140余万人。五是健全儿童用药供应保障机制。印发《[关于保障儿童用药的若干意见](https://www.pkulaw.com/chl/c8a7d2f29c68fddcbdfb.html)》，先后发布3批共105个鼓励研发申报儿童药品，通过“重大新药创制”科技重大专项投入5.74亿元，支持儿童药品研发和临床研究。加强儿童用药重点监测，鼓励儿童药品集中生产基地建设。六是持续加强儿童健康科研投入。国家科技计划部署儿童生长发育与疾病防治相关经费由“十二五”时期的7561万元，增长到“十四五”时期的1.26亿元。推进儿童健康领域科技创新平台建设，强化儿童健康领域临床医学研究中心和重点实验室建设。
　　（三）建设防治结合的儿童健康服务体系。认真落实《关于加强儿童医疗卫生服务改革与发展的意见》，不断加大改革力度。一是加强儿童医疗服务网络建设。设置2个国家儿童医学中心和5个国家儿童区域医疗中心，有序推进儿科方向国家医学中心和国家区域医疗中心项目建设，持续加强儿科相关国家临床重点专科建设，构建国家、区域、省、市、县级儿童医疗服务网络。2013-2021年，累计安排中央预算内投资309亿元支持全国1960个儿童医院和妇幼保健机构建设。截至2021年底，我国共有儿童医院151家，每千名儿童床位数达2.2张，较2015年增加0.27张。二是强化儿童保健服务网络建设。构建以省、市、县三级妇幼保健机构为核心、基层医疗卫生机构为基础的儿童保健服务网络。2019-2021年，中央财政累计安排27.91亿元支持省级和县级妇幼保健机构能力建设。2021年，全国共有妇幼保健机构3032家，全年总门诊量3.31亿人次，乡镇卫生院3.5万家，社区卫生服务中心（站）3.6万家，当年提供儿童健康体检8550万人次。三是加快儿童健康人才队伍建设。引导和支持高校新增27个儿科学、1个中医儿科学专业布点，加强“5＋3”高校一体化儿科硕士专业学位研究生招收培养。加大儿科等紧缺专业住院医师规范化培训招收，大力开展转岗培训和继续医学教育，实施中西部儿童保健人员培训项目。印发《[关于深化公立医院薪酬制度改革的指导意见](https://www.pkulaw.com/chl/538fea45969d753ebdfb.html)》，推动薪酬待遇政策向儿科医务人员倾斜。我国儿科执业（助理）医师数从2015年的11.8万人，增加到2021年的20.6万人。四是推进儿童保健与临床服务有机融合。突出以儿童健康为中心，打破妇幼保健机构“防”和“治”的科室分设格局，优化服务流程，整合服务内容，强化中医药服务，改革组建孕产保健部、儿童保健部和妇女保健部，努力构建系统连续、防治结合的服务新模式。五是推动儿童健康服务体系上下联动。推动基层医疗卫生机构、妇幼保健机构与专科医院建立儿童疾病筛查、转诊工作机制，落实分级诊疗制度，强化医疗联合体建设，促进优质资源下沉基层。六是促进儿童健康信息互联互通。推进“互联网＋妇幼健康”发展，实施“云上妇幼”项目，促进上下互联、横向互通、方便快捷、智慧服务。以推进出生医学证明在线申领和电子证照应用为抓手，积极推进“出生一件事”跨部门、跨地区办理，逐步实现“网上办”“掌上办”，极大方便了群众。
　　（四）推动形成促进儿童健康的协同支持体系。一是推动形成儿童友好的社会环境。优化城市公共空间规划，推动全社会关心关爱儿童健康成长。二是加大家庭养育支持力度。设立3岁以下婴幼儿照护个人所得税专项附加扣除，按照每个婴幼儿每月1000元的标准定额扣除。实施母乳喂养促进行动计划，推进婴幼儿营养喂养咨询指导，保护女职工权益，推进公共场所和用人单位母婴设施建设，全力支持母乳喂养。促进托育服务健康发展，严格托育机构标准设置和规范管理，开展普惠托育专项行动。三是持续加大环境保护和治理力度。以最严标准推进生态保护和环境治理，淘汰升级落后产能，推广使用清洁能源，儿童群体性铅中毒事件明显减少。四是加强食品安全监管。严格落实“四个最严”要求，建立完善食品安全标准体系，连续5年将婴幼儿配方食品、辅助食品作为高风险品种列入食品安全监管重点，加大抽检和执法力度。印发《[学校食品安全与营养健康管理规定](https://www.pkulaw.com/chl/832d69728649a0cebdfb.html)》，推进中小学、幼儿园建立集中用餐陪餐制度，对校园食堂等集中供餐单位开展风险排查，大力推进学校食堂“明厨亮灶”工作，截至2021年底，全国学校食堂“明厨亮灶”覆盖率达99.8%。五是加大儿童用品质量监督抽查和执法力度。连续3年开展儿童和学生用品安全守护行动，共检查儿童用品生产企业2.4万家、重点商超和批发市场25.4万家，监督抽查产品5.4万批次，立案查处违法案件0.5万起，严格实施童车、玩具、儿童安全座椅强制性产品认证。六是加大对儿童产品市场反不正当竞争执法力度。连续5年开展反不正当竞争执法专项行动，聚焦近视眼镜市场、校外培训、早教机构等重点领域，严厉打击虚假宣传等侵害儿童合法权益的不正当竞争行为。七是进一步减轻义务教育阶段学生作业负担和校外培训负担。坚持健康第一的教育理念，强化德智体美劳全面培养，指导学校提高作业管理、课后服务和课堂教学质量，严格校外培训管理，给孩子留出更多运动时间。据调查，73%的家长表示孩子完成书面作业时间比“双减”前明显减少。八是大力促进儿童体育运动。全面加强和改进新时代学校体育工作，深化体育教学改革，不断加大学校体育课比重。开展儿童青少年体育活动促进，针对儿童青少年近视、肥胖、脊柱侧弯等进行体育干预，在社区、公园、绿地等公共场所加强配备适合未成年人身体锻炼的体育设施。“十三五”期间，每年利用中央资金9亿多元，支持1000多个公共体育场馆向青少年等人群免费或低收费开放。
　　（五）强化儿童健康全过程全方位服务。一是强化生命早期1000天健康保障。加强全方位孕产期保健，实施母婴安全五项制度，加强危重孕产妇、危重新生儿救治中心建设，保障生育政策调整完善。2012年以来，全国住院分娩率保持在99%以上，县级危重新生儿救治中心覆盖率达90.7%。二是扎实推进出生缺陷三级防治措施落实。实施免费孕前优生健康检查，产前筛查率提高至81.1%，新生儿遗传代谢病和听力障碍筛查率保持在90%以上。安排中央专项彩票公益金实施出生缺陷干预救助项目，救助困难家庭患儿。2021年启动实施罕见病诊疗能力水平提升项目，进一步推进儿童罕见病救治。三是加强7岁以下儿童健康管理。在全国推广使用《母子健康手册》，规范生育全程基本医疗保健服务，普及科学育儿知识，促进儿童早期发展。2021年，全国7岁以下儿童健康管理率为94.6%。四是着力推进儿童营养改善。印发《国民营养计划（2017-2030年）》，开展相关重大行动。2011年以来实施农村义务教育学生营养改善计划，中央财政累计安排补助资金1968亿元，每年惠及约3800万学生。2012年以来持续开展贫困地区儿童营养改善项目，中央和地方财政累计投入52亿元，受益儿童1365万。五是加强幼儿园和学校卫生保健。将健康教育纳入国民教育体系，大力推进青春期保健服务，加强监督指导，提高儿童青少年疾病防控意识和生殖健康水平。修订印发《[中小学生健康体检管理办法](https://www.pkulaw.com/chl/dc6889891d10f4e8bdfb.html)》，规定中小学校每年组织1次在校学生健康体检，学生体检率持续提高。
　　（六）集中力量解决儿童健康重点问题。一是综合防控儿童青少年近视。全面加强儿童青少年近视防控，制定发布《综合防控儿童青少年近视实施方案》，持续开展近视监测，开展儿童青少年近视防控适宜技术和中医适宜技术试点，加强儿童在新冠肺炎疫情期间近视预防专业指导，强化学校卫生监督检查，积极推进近视防控产品违法违规商业宣传整治。各级政府逐级签订工作责任书，加强评议考核，以最严的措施、最大的力度加强近视防控，努力让孩子们拥有一个光明的未来。2020年，0-6岁儿童眼保健和视力检查覆盖率91.8%；儿童青少年总体近视率52.7%，比2018年下降0.9个百分点。二是加强儿童常见传染病防治。统筹做好国家免疫规划疫苗常规免疫工作，以乡镇为单位国家免疫规划疫苗接种率维持在90%以上。持续实施艾滋病、乙肝母婴阻断，艾滋病母婴传播率下降到2021年的3.3%，降至历史最低点。三是加大儿童重大疾病救治力度。加强综合医疗保障，开展集中定点救治，逐步扩大救治病种范围，将免疫性溶血性贫血、中枢神经系统肿瘤等12个病种纳入儿童血液病、恶性肿瘤救治管理病种范围。2013-2021年，中央财政通过彩票公益金预算安排16.6亿元专项救助贫困白血病和先心病儿童。2021年，全国儿童肿瘤患者省域内就诊率达到85%以上。四是加强儿童青少年肥胖防控。印发《儿童青少年肥胖防控实施方案》，加大科学指导和科普宣教力度，加强监测和评价，持续开展重点地区营养干预项目，推广合理膳食和科学运动等理念，家校联动、社会联手，推动儿童“管住嘴、迈开腿”。五是促进儿童青少年心理健康。将儿童青少年心理健康纳入健康中国行动统筹推进，强化学校心理健康工作人员配备，推动精神卫生服务网络建设。以抑郁症、孤独症为重点，探索防治适宜技术和干预模式。全国1379家妇幼保健机构开展儿童心理保健服务，318个地市设有精神专科医院，2756个区县能够开展精神卫生专科服务，初步缓解了基层儿童心理服务短缺问题。
　　（七）加强新冠肺炎疫情期间儿童健康保障。坚持人民至上、生命至上，在疫情防控中对儿童予以重点救治保障。一是积极预防儿童新冠肺炎。普及疫情防控知识，规范儿童口罩生产和佩戴，指导学校和家长做好儿童防控措施落实。大力推广线上健康评估和指导等智慧服务。指导各级各类医疗机构做好发热患儿筛查和疫情防控工作。二是全力保障孕产妇安全分娩。指导各地向社会公布孕产妇产检和住院分娩定点医院，建立危重孕产妇、新生儿救治绿色通道。做好危重感染孕产妇救治工作，保障疫情期间孕产妇安全分娩，减少新生儿感染。三是稳妥推进儿童新冠病毒疫苗接种。周密组织安排，强化医疗保障，全力做好新冠病毒疫苗供应，切实做到儿童安全接种和应接尽接。全国3-17岁人群新冠病毒疫苗第一剂次接种覆盖率和全程接种覆盖率分别达到98%和95%以上。四是加强儿童救治。及时更新《新型冠状病毒肺炎诊疗方案》中儿童患者诊疗内容，强化中医药防治。按照“四集中”原则，加强儿童定点救治，针对儿童患病特点，指导各地加强儿童心理疏导和人文关爱，努力保障儿童生命安全和身心健康。
　　党的十八大以来，在以习近平同志为核心的党中央坚强领导下，我国儿童健康工作投入力度持续加大，政策体系不断完善，儿童健康水平整体明显提高，《中国儿童发展纲要（2011-2020年）》的儿童健康相关目标均如期实现。2021年全国婴儿死亡率、5岁以下儿童死亡率分别为5.0‰和7.1‰，较2012年下降51.5%和46.2%，总体优于中高收入国家平均水平。6岁以下儿童生长迟缓率和低体重率等指标逐步改善，特别是农村儿童生长迟缓问题改善明显。儿童常见传染病得到有效控制，2021年0-18岁儿童法定传染病报告发病率为928.16/10万，较2012年下降16.2%。严重高发出生缺陷得到初步控制，与2010年相比，2020年出生缺陷导致5岁以下儿童死亡率由2.8‰降至1.3‰，神经管缺陷、唐氏综合征等严重致残出生缺陷发生率降幅达41%。2020年，18岁以下儿童伤害死亡率由2010年的22.41/10万降至11.06/10万，降幅50.6%。

　　**二、**存在的主要问题
　　（一）从儿童健康服务需求看。一是区域、城乡间儿童健康状况不平衡。2021年，农村婴儿死亡率是城市的1.8倍，西部地区是东部地区的2.2倍；农村5岁以下儿童死亡率是城市的2.1倍，西部地区是东部地区的2.2倍。二是儿童健康主要影响因素逐步转变。我国已消除新生儿破伤风，5岁以下儿童死亡率持续下降，但一些影响儿童正常生长发育的健康问题凸显。儿童青少年总体近视率仍处高位，超重问题不容忽视，脊柱侧弯、孤独症患病增多，溺水、道路交通事故等伤害是5岁以下儿童的主要死因。儿童肿瘤、心脏病等慢性非传染性疾病及罕见病等依然威胁儿童健康。三是新冠肺炎疫情发生以来，儿童居家和上网课增多，体育运动减少，在电子屏幕前暴露时间延长，导致较多儿童青少年出现近视加重情况。四是科学养育方面问题仍较突出。家庭科学育儿知识总体不足，“隔代养育”现象普遍，城市中长辈过度溺爱儿童娇生惯养，农村留守儿童缺乏父母关爱。同时，群众对儿童健康提出更高要求，希望儿童得到更高水平的医疗保健服务，食品玩具更安全、体育运动更丰富、社会保障更健全。
　　（二）从儿童健康服务供给看。一是儿童医疗卫生资源总量不足与分布不均衡问题并存，优质资源主要分布在东部和城市地区，中西部地区和农村地区优质资源较为薄弱。二是我国儿童保健服务网络特别是基层儿童保健服务网络有待加强。据调查，基层配备专职人员从事儿童保健服务的乡镇卫生院数量仅占总数的52.2%，难以满足日益增长的儿童保健需求。三是儿童医疗服务人才短缺且工作负担重，吸引力不强。我国每千名儿童儿科执业（助理）医师数与发达国家相比仍有较大差距，儿童心理、康复等特殊领域人才紧缺。虽然儿科医师总体数量有所提高，但近年来在总体医师中的占比变化不大，人才队伍发展相对缓慢。四是群众获取科学育儿知识的渠道仍需完善，相关媒体、社会组织和学校等健康教育和社会宣传力度有待进一步加大。五是儿童健康促进支持政策有待加强。托育服务尚不能满足群众实际需求；学校校医配备有缺口，能力水平有待进一步提高；儿童食品用品安全执法需持续加强；“双减”政策下，儿童青少年参与体育运动需求增多，体育场地设施等仍然不足。
　　（三）从儿童健康保障机制看。一是儿童健康法制保障需要进一步加强，儿童用药、儿童心理健康、儿童伤害防控等方面还存在制度短板。二是儿童健康投入机制需要进一步完善，以更好适应儿童健康需求，推动解决预防投入不足等问题。三是儿童健康领域相关改革还需持续深化。相关医疗机构特别是综合医院、妇幼保健机构和基层医疗机构，因儿童所需的药品、耗材品种繁多，人员和运行成本高，业务收入少，发展儿科和儿童保健科动力不足，对于儿科医务人员的倾斜政策在部分地区和医疗机构尚未落实到位。四是儿童用药总体规模较小，临床试验难度大，研发成本高，企业研发生产动力不强，儿童用药适宜剂型、适宜规格依然有限，小品种药仍存在供应风险。五是儿童健康促进需要各有关部门密切配合，全社会共同参与，特别是涉及儿童伤害预防、食品安全、学校卫生、儿童心理健康、肥胖干预、健康教育宣传等问题，需要进一步加大力度协同推进。

　　**三、**下一步工作安排
　　坚持以习近平新时代中国特色社会主义思想为指导，深入贯彻习近平总书记关于儿童健康工作的重要指示精神，认真落实党中央、国务院决策部署，坚持以人民健康为中心，强化大卫生大健康理念，依法行政、强化保障、创新机制、强基固本，不断推进儿童健康事业高质量发展。
　　（一）强化儿童健康促进法制建设。一是进一步完善儿童健康法律法规体系。积极争取将儿童健康促进工作急需的立法修法项目列入下一届全国人大常委会五年立法规划。尽快启动修订[学校卫生工作条例](https://www.pkulaw.com/chl/a6c3bf030ff634f1bdfb.html)，研究完善儿童健康促进工作相关法规和配套规定。二是持续推动儿童健康促进相关法规政策落实。以法制保障儿童健康，进一步推动各级政府和各有关部门落实儿童健康促进主体责任。将儿童优先发展放在重要位置，在制定经济社会发展规划中优先考虑，在公共资源配置上优先满足，建立稳定的与经济社会发展相协调的儿童健康投入保障制度。
　　（二）深化儿童健康领域改革创新。一是加强儿童医疗保障。逐步提高儿童基本医疗保障水平，统筹发挥基本医保、大病保险、医疗救助三重制度综合保障梯次减负作用，共同减轻患儿医疗费用负担。落实落细参保缴费政策，确保儿童应保尽保。充分发挥商业保险风险保障的补充功能，鼓励商业保险机构开发针对儿童的商业健康保险产品。二是稳妥有序推进深化医疗服务价格改革试点，指导试点城市按照改革思路和机制设计完善本地实施方案，在机制设计上将支持儿科医疗服务发展作为相关指标统筹考虑。三是深化公立医院薪酬制度改革。切实落实保障儿科和儿童保健科医务人员薪酬待遇的倾斜政策，吸引更多优秀人才从事儿童健康事业。四是强化儿童用药保障。进一步加大对儿童药品研发生产等方面政策支持力度，完善儿童用药监测预警机制，持续推动儿童药品集中生产基地建设，扩大品种覆盖，深化供应链协作，努力推动实现一批儿童国产药品上市。动态调整国家医保药品目录，支持将符合条件的儿童药品纳入国家医保药品目录，定期开展国家医保药品目录准入谈判，支持将符合条件的罕见病用药等儿童药品纳入谈判范围。五是强化儿童健康科技创新支撑。大力支持儿童重大疾病预防、诊断、治疗、康复和健康管理等方面科技研发，加强科技创新体系建设，促进成果转化惠及儿童健康。
　　（三）健全儿童医疗卫生服务体系。一是推进儿童医疗卫生服务高质量发展。“十四五”期间，继续安排中央预算内投资支持儿科项目建设，支持开展10个左右儿科类国家区域医疗中心建设项目，推动儿科优质医疗资源扩容和均衡布局，推进优质资源下沉。进一步筑牢基层儿童保健服务网底，着力提高乡镇卫生院、社区卫生服务中心和村卫生室儿童保健服务能力。根据需要完善儿童医院建设相关标准，开展母婴友好医院和儿童友好医院建设。二是加快开展重点人群健康服务补短板工作。通过中央预算内投资，实施妇女儿童健康服务能力建设工程，支持每省份1个省级儿科项目建设，以及分娩量较大、人口较多的地市级妇幼保健机构项目建设。支持革命老区、民族地区、边疆地区和欠发达地区的儿童健康服务体系发展。三是加强儿童健康人才队伍建设。加强不同层次儿科医学人才培养，重点培养儿童保健、营养、心理、康复等紧缺专业人才。推进实施基层医疗卫生机构全科医生特设岗位计划，每所乡镇卫生院、社区卫生服务中心至少配备1名全科医生提供规范的儿童基本医疗服务，至少配备2名专业从事儿童保健的医生，努力补齐人才短板。
　　（四）加强儿童健康全程服务。一是把好生育关，围绕孕期、产前、分娩期、新生儿期等儿童的生命最早期，加强孕产期保健、母婴安全保障和出生缺陷防治等，着力提高孕产期服务质量。二是把好养育关，主要围绕儿童营养喂养、交流玩耍、生活照护、伤害预防等，加强对养育人的健康指导，促进儿童早期发展。三是把好发育关，主要围绕儿童体格生长发育和神经心理发育，加强儿童保健服务，开展系统化儿童健康综合管理和体质锻炼指导，健全儿童心理健康服务体系，强化儿童心理保健和干预，提高儿童环境和社会适应能力。四是把好教育关，强化幼儿园、托育机构卫生保健工作，加强学校心理教师和校医及幼儿园保健医配备，科学规划新冠肺炎疫情期间课程安排，强化学生健康教育，从小抓起，引导儿童树立正确健康观念，养成良好行为习惯。五是把好疾病防治关，围绕儿童传染病、常见病、多发病，以及近视、肥胖、脊柱侧弯、孤独症、心理健康等问题，进一步完善防控体系和工作机制，全面实施残疾儿童康复救助制度，做好儿童新冠肺炎防治，切实做到预防为主、防治结合。
　　（五）强化儿童健康促进工作协同。一是强化政策统筹。加强中国儿童发展纲要落实。结合推进公共服务补短板、强弱项、提质量，促进公共资源向儿童适度倾斜。二是落实家庭支持政策。大力推进[家庭教育促进法](https://www.pkulaw.com/chl/99c5277887339564bdfb.html)实施，引导全社会注重家庭、家教、家风，将健康促进和科学育儿纳入家庭教育。加快普惠托育服务发展，到2025年实现每千人口拥有3岁以下婴幼儿托位数达4.5个。完善生育休假制度，健全假期成本合理分担机制，保障妇女就业和劳动权益，落实税收等支持政策，减轻家庭养育负担。三是落实完善儿童教育、体育相关政策。深入推进“双减”政策落实，规范校外培训，增加体育和劳动等课程。开展青少年科学健身指导普及活动，突出体教融合，支持学校全覆盖、高质量开展体育类课后服务，加强公共体育设施供给，推动体育、文化场馆向儿童青少年免费开放。四是完善社会关爱政策，开展儿童健康综合发展示范县（市、区、旗）创建，在城市规划中引入“1米高度看城市”的儿童视角，将儿童健康促进纳入城市更新行动，在交通管理、公园绿地布局等方面更多考虑儿童身心健康和发展需要。加强儿童食品、玩具、用品等生产、销售全程监管，持续加大执法力度。建立健全多部门参与的预防儿童伤害工作机制，完善儿童伤害监测报告系统。五是充分发挥妇联、共青团、计生协等群团组织作用，加强国际交流合作，共同做好儿童健康政策宣教实施。充分调动社会组织和企业积极性，汇聚各方力量保障儿童健康。
　　我们将更加紧密地团结在以习近平同志为核心的党中央周围，坚持以习近平新时代中国特色社会主义思想为指导，在全国人大及其常委会的监督支持下，进一步做好儿童健康促进工作，深入推进儿童健康事业改革发展，不断提升儿童健康水平，以实际行动迎接党的二十大胜利召开！

© 北大法宝：（[www.pkulaw.com](https://www.pkulaw.com/)) 专业提供法律信息、法学知识和法律软件领域各类解决方案。北大法宝为您提供丰富的参考资料，正式引用法规条文时请与标准文本核对。
欢迎查看所有[产品和服务](https://www.pkulaw.com/helps/2.html)。法宝快讯：[如何快速找到您需要的检索结果？](https://www.pkulaw.com/helps/70.html)[法宝](https://www.pkulaw.com/helps/69.html)V6有何新特色？

扫描二维码阅读原文

**原文链接：**<https://www.pkulaw.com/chl/12cb9cf195be1fb3bdfb.html>
